# Nicotine use during late adolescence and young adulthood is associated with changes in hippocampal volume and memory performance

**DOI:** 10.3389/fnins.2024.1436951

**Published:** 2024-08-16

**Authors:** Joseph P. Happer, Kelly E. Courtney, Rachel E. Baca, Gianna Andrade, Courtney Thompson, Qian Shen, Thomas T. Liu, Joanna Jacobus

**Affiliations:** ^1^Department of Psychiatry, University of California, San Diego, San Diego, CA, United States; ^2^Department of Neurosciences, University of California, San Diego, San Diego, CA, United States; ^3^Center for Functional MRI, University of California, San Diego, San Diego, CA, United States; ^4^Department of Radiology, University of California, San Diego, San Diego, CA, United States

**Keywords:** hippocampus, memory, nicotine, vaping, adolescence, young adult

## Abstract

**Background:**

With the advent of electronic nicotine delivery systems, the use of nicotine and tobacco products (NTPs) among adolescents and young adults remains high in the US. Use of e-cigarettes additionally elevates the risk of problematic use of other substances like cannabis, which is often co-used with NTPs. However, their effects on brain health, particularly the hippocampus, and cognition during this neurodevelopmental period are poorly understood.

**Methods:**

Healthy late adolescents/young adults (*N* = 223) ages 16–22 completed a structural MRI to examine right and left hippocampal volumes. Memory was assessed with the NIH Toolbox Picture Sequence Memory Test (PSMT) and Rey Auditory Verbal Learning Test (RAVLT). Cumulative 6-month NTP and cannabis episodes were assessed and modeled continuously on hippocampal volumes. Participants were then grouped based on 6-month NTP use to examine relationships with the hippocampus and memory: current users (CU) endorsed weekly or greater use; light/abstinent users (LU) endorsed less than weekly; and never users (NU).

**Results:**

NTP use predicted larger hippocampal volumes bilaterally while cannabis use had no impact nor interacted with NTP use. For memory, larger left hippocampal volumes were positively associated with PSMT performance, RAVLT total learning, short delay and long delay recall for the NU group. In contrast, there was a negative relationship between hippocampal volumes and performances for LU and CU groups. No differences were detected between NTP-using groups.

**Conclusion:**

These results suggest that the hippocampus is sensitive to NTP exposure during late adolescence/young adulthood and may alter typical hippocampal morphometry in addition to brain-behavior relationships underlying learning and memory processes.

## 1 Introduction

Rates of nicotine/tobacco-related product (NTP) use remain high among U.S. adolescents and young adults despite declining use of traditional combustible cigarettes (Gentzke, 2019). With the advent of electronic nicotine delivery systems, vaping has dramatically increased over the past several years (Johnston et al., 2020). Roughly 21% of 12th graders and approximately 14% of young adults reported e-cigarette usage within the past month in recent studies (Miech et al., 2023; Patrick et al., 2023). Vaping allows for easy consumption across the day often leading to increased use and intensity (Cerdá et al., 2020; Vogel et al., 2020). This is particularly concerning among adolescents who may be more vulnerable to nicotine dependence even after minimal exposure (DiFranza et al., 2000, 2007; Lin et al., 2022). Use of e-cigarettes has also been associated with increased risk for problematic use of other substances such as cannabis (Cobb et al., 2018; Fadus et al., 2019), which is also commonly co-used with NTPs (Knapp et al., 2019; Moustafa et al., 2022). Indeed, with reports of NTP and cannabis co-use among late adolescents and young adults ranging anywhere from 21% to over 50% in the past month (Cobb et al., 2018; Dunbar et al., 2020; McCauley et al., 2024), understanding the effects of NTPs in the context of cannabis use is essential, particularly as state and local laws may influence patterns of co-use (Wang et al., 2016).

Adolescence is a critical period of neurodevelopment that extends into early adulthood, marked by significant changes in gray matter tissue. This phase involves the maturation of cortical and subcortical brain structures through processes including synaptic pruning and cortical thinning (Spear, 2013; Wierenga et al., 2014). Use of substances such as nicotine during this period may alter these developmental trajectories (Akkermans et al., 2017; Chaarani et al., 2019). Studies using structural magnetic resonance imaging (MRI) to examine brain health in nicotine-using adolescents/young adults have noted that nicotine use in younger populations has been associated with changes in cortical regions, such as reduced thickness in the medial prefrontal, insular, parahippocampal, and temporal regions (Li et al., 2015; Dai et al., 2022; Hernandez Mejia et al., 2024). Alterations in subcortical regions have also been noted, including smaller amygdala and thalamus volumes and larger striatum volumes (Li et al., 2015; Yu et al., 2018). These changes in adolescent brain structure have been observed even after minimal nicotine exposure (Chaarani et al., 2019), and animal models suggest that low, intermittent doses of nicotine during adolescence can have a lasting impact on brain health and functioning that continues later into adulthood (Trauth et al., 2000; Abreu-Villaça et al., 2003; Oliveira-da-Silva et al., 2009; Leslie, 2020). However, few studies have examined the structural integrity of the hippocampus in NTP-using adolescents and young adults, and those that have report smaller volumes in NTP users (Harper et al., 2023) or no differences (Filbey et al., 2015) compared to non-users. Notably, these studies were conducted in young adults who primarily smoked traditional combustible cigarettes, which likely result in greater toxic exposure compared to the e-cigarettes more commonly used today (Rubinstein et al., 2018; Marques et al., 2021; Lin et al., 2022; Wade et al., 2022).

Within neurobiological models of substance use and addiction, the hippocampus is heavily implicated in the development and maintenance of substance use disorders (Volkow et al., 2019) as it modulates reinforcement learning and episodic memory of rewarding stimuli (Subramaniyan and Dani, 2015), and these processes may be especially heightened in adolescents (Yuan et al., 2015). Adolescents and young adults who regularly use NTPs have demonstrated impairments on measures of hippocampal-dependent processes, such as working memory, verbal memory, and attention compared to their non-using counterparts (Jacobsen et al., 2005, 2007a,b,c; Fried et al., 2006; Colby et al., 2010; Filbey et al., 2015; Treur et al., 2015; Wade et al., 2021; Dai et al., 2022). Functional neuroimaging studies have similarly found alterations in hippocampal and parahippocampal activation elicited by working memory (Jacobsen et al., 2007a,b) and smoking-related cues (Rubinstein et al., 2011; Chen et al., 2018) in NTP-using adolescents. Importantly, nicotine binds to nicotinic acetylcholine receptors (nAChRs), which are found in abundance throughout the hippocampus (Zeid et al., 2018), and stimulation of these nAChRs may enhance synaptic connections with other regions involved in addiction such as the nucleus accumbens (Subramaniyan and Dani, 2015). Hippocampal structure (Lorenzetti et al., 2019) and functioning (Filbey et al., 2015; Scott et al., 2018; Jacobus et al., 2019) are also impacted in cannabis-using adolescents as endocannabinoid receptors are also widely distributed in the hippocampus (Mechoulam and Parker, 2013); yet, few studies have examined how cannabis-related hippocampal alterations may also be modulated by nicotine co-use (Filbey et al., 2015). Thus, it is important to consider the impact of NTP use on hippocampal integrity in the context of cannabis co-use, given the high prevalence of co-use among adolescents/young adults (Cobb et al., 2018).

In light of these considerations, the aim of the current study was to examine the associations between recent NTP use, primarily in the form of e-cigarette use, and bilateral hippocampal volume estimates in a sample of adolescent and young adults aged 16–22. Additionally, it was investigated whether cannabis use mediates the relationship between NTP use and hippocampal volume estimates in this age group. Relationships between nicotine use, hippocampus volumes, and performances on measures of verbal and non-verbal learning and memory were also investigated. It was hypothesized that greater cumulative NTP use would be associated with smaller hippocampal volumes, and nicotine would negatively impact hippocampal-based memory assessments.

## 2 Methods

### 2.1 Participants and procedures

Two hundred and twenty-three participants ages 16–22 were recruited as part of a study on the effects of nicotine and cannabis co-use on brain structure and function during adolescence/young adulthood. As previously reported (Courtney et al., 2020, 2022), participants were recruited via flyers posted physically and electronically at schools, community colleges, four-year universities, and social media sites targeting San Diego County. Initial recruitment was stratified based on use of NTP, cannabis products, or both during the previous 6-month period to ensure variability in NTP and cannabis use. For the purposes of examining the relationship between hippocampal volume and memory performances, participants were categorized into three groups based on 6-month NTP frequency alone: Current Users (CU) who endorsed ≥26 NTP use episodes (~at least weekly); Light/Abstinent Users (LU) who endorsed <26 use episodes (< weekly use); and Never Users (NU) who endorsed having never used NTP in their lifetime. NTP use was defined as the use of any combination of electronic cigarettes (e.g., vape pens, e-hookah), combustible cigarettes, hookah with tobacco, tobacco pipe, cigars (including blunts, spliffs), snus, smokeless tobacco, chew, snuff, and/or nicotine replacement.

Exclusion criteria included >10 lifetime episodes of illicit substance use; lifetime DSM-5 psychiatric diagnoses other than tobacco and/or cannabis use disorder; acute influence of cannabis or alcohol use at study visit; use of any psychoactive medications; major medical problems; MRI contraindications; or history of prenatal substance exposure or developmental disability.

Participants completed a single 4-h session consisting of a battery of interviews, self-report assessments covering demographic information, mental health, substance use, and neurocognitive functioning, which was followed by an MRI session. Before beginning the study session, all participants gave written informed consent (≥18 years old) or parental consent and participant assent (<18 years old). Participants were asked to refrain from using cannabis and alcohol 12 h prior to the appointment, which was confirmed with oral fluid, urine, and breathalyzer. Urine samples were used to confirm abstinence from illicit substances. Participants abstained from caffeine for at least 30 min prior to MRI scanning. They were not required to abstain from NTP use to avoid nicotine withdrawal effects during testing. Time of last NTP use was documented. All procedures were approved by the University of California, San Diego Human Research Protections Program.

### 2.2 Measures

Demographic data (e.g., age, sex at birth, race/ethnicity, education) were obtained from a psychosocial interview. To assess quantity and frequency of NTP and cannabis use, the Customary Drinking and Drug Use Record structured interview (Brown et al., 1998) was used, including a modification to include additional nicotine and cannabis questions (Jacobus et al., 2018; Karoly et al., 2019a,b). Past 6 months and lifetime use were measured in terms of independent episodes, allowing for multiple uses to be reported within a single day (e.g., first thing in the morning, again before bed). Participants were asked to provide additional details related to each substance reported including age at first use and onset of regular (weekly) use. Alcohol use was queried for the previous 30 days. For this study, individuals who reported having never used a substance (i.e., NTP, cannabis, alcohol) were recorded as having zero episodes during the respective timeframes.

### 2.3 Memory assessment

As part of a comprehensive neurocognitive battery, participants completed the Picture Sequence Memory Test (PSMT) from the National Institutes of Health (NIH) toolbox cognition battery (Weintraub et al., 2013) and the Rey Auditory Verbal Learning Task (RAVLT, Schmidt, 1996). For this study, the primary test of interest from the NIH toolbox was the PSMT; however, participants completed all seven cognitive tasks of the toolbox battery, which were later used to compute crystalized composite scores (derived from Picture Vocabulary and Oral Reading tests). The PSMT is a measure of episodic memory where participants had to recall a sequence of delayed pictures. The task was completed using the NIH Toolbox app on 3^rd^ generation iPad Air devices (10.5 in). Participants were seated upright and used their dominant index finger to make each response. To prevent participants from inadvertently skipping through instructions, a one-second touch-and-hold button was required to advance to the next task. Population-adjusted scores, which adjusted for age, sex, race, ethnicity, and education level, were used in the present analyses.

For the RAVLT, participants complete five learning trials during which they were read a list of 15 words and asked to recall the list at the end of each trial. A new, second list was read, and then participants were asked to again recall the original list after this short delay. After 30 min, they were again asked to recall the original list. Total raw score over all learning trials, short delay recall, and long delay recall were examined.

### 2.4 Imaging acquisition and processing

Participants were scanned on a 3.0 Tesla GE Discovery MR750 scanner with a 32-channel receive head coil at the UCSD Center for Functional MRI. A high-resolution T1-weighted anatomical fast spoiled gradient echo (FSPGR) scan was acquired with TI/TE/TR = 1060/2/2500 ms, 256×256 matrix, flip angle = 8°, FOV = 256 mm, 1.0 mm^3^ voxels. Brain images for each participant were spatially normalized, field-bias corrected, and segmented using the Freesurfer pipeline (version 6.0, Fischl et al., 2002, 2004). Right and left hippocampal volumes and an estimate of total brain volume (“BrainSegVolNotVent”) were extracted for analyses.

### 2.5 Data analyses

#### 2.5.1 Hippocampal volume

Data analyses were conducted using R (v4.3.2). Changes in bilateral hippocampal volumes were examined using linear regressions that modeled cumulative 6-month NTP use episodes, cumulative 6-month cannabis use episodes, and their interaction as continuous variables, while controlling for demographic factors (i.e., age, sex), past 30-day alcohol use, and estimated total brain volume as covariates in the model. Follow-up analyses explored potential moderators of this relationship including age of NTP initiation and recency of NTP use within those who reported lifetime NTP use. Combustible cigarette usage was similarly explored as a possible moderator given the potential for greater toxic exposure and addiction severity (Rubinstein et al., 2018; Marques et al., 2021; Lin et al., 2022; Wade et al., 2022).

The large range of both cannabis and NTP cumulative use episodes raises the possibility of a single or several data points having significant leverage on the models (Belsley et al., 2005). If a substance use variable was significant within the model, the variable was examined for influential points using DFBETAS. Highly influential points were defined as those whose DFBETAS were above the calculated threshold (2÷√*n*) of 0.135 (Belsley et al., 2005). Models were then rerun without those data points.

#### 2.5.2 Hippocampal volume and memory performance

Participants were then grouped based on NTP status as described above (i.e., Current Users [CU], Light/Abstinent Users [LU], and Never Users [NU]) to examine relationships with hippocampal volume and memory. Group characteristics were compared using ANOVA and chi-square tests for continuous and categorical variables, respectively. The contribution of NTP group status, left and right hippocampal volumes, and their interaction to demographically-adjusted T-scores from the PSMT were examined using individual linear regressions for each hemisphere, controlling for NIH toolbox crystalized composite scores, 6-month cannabis use, and 30-day alcohol use, which were modeled as continuous covariates in the model. Raw RAVLT Total Learning, Short Delay, and Long Delay were similarly examined as outcomes with individual linear regressions, controlling for age, sex, crystalized composite score, cannabis use, and alcohol use as covariates in the model. A statistically significant threshold of *p* < 0.05 was set for all analyses.

## 3 Results

### 3.1 Participants

The final sample (*N* = 223) was roughly split on sex at birth (54% male) and approximately 50% self-identified as White (see Table 1). Between groups comparisons indicated that relative to both NTP use groups, NU were younger (*p*’s < 0.001), had fewer years of education (*p*’s < 0.01), fewer alcohol use episodes over the past 30 days (*p*’s < 0.0001), and fewer cannabis use episodes over the past 6 months (*p*’s < 0.0001). Additionally, NU had more females compared to CU (*χ^2^* = 6.8, *p* = 0.009), while there were no differences in sex between CU and LU (*p* > 0.07). The NTP groups were found to differ only on NTP use episodes in the past 6 months (*t* = −5.3, *p* < 0.0001) and days since last NTP use (*t* = 4.5, *p* < 0.001), as anticipated.

**Table 1:** Sample demographics and characteristics.

	Never Users^a^ *N* = 67^1^	Light/Abstinent Users^b^ *N* = 64^1^	Current Users^c^ *N* = 92^1^	*p*-value^2^
Age	18.8 (±1.7)^b,c^	19.8 (±1.5)^a^	19.8 (±1.5)^a^	<0.001
% Male	29 (43%)^c^	32 (50%)	59 (64%)^a^	0.026
Race/Ethnicity				
% White	29 (43%)	32 (50%)	51 (55%)	0.11
% Hispanic	28 (42%)	28 (44%)	29 (32%)	0.2
Education (Years Completed)	12.6 (±1.7)^b,c^	13.3 (±1.3)^a^	13.3 (±1.4)^a^	0.003
NIH Toolbox Crystalized Composite (Age-Corrected)	106 (±15)	108 (±11)	105 (±11)	0.3
Alcohol Use Previous 30 Days	2.0 (±3.6)^b,c^	5.6 (±4.8)^a^	6.7 (±5.5)^a^	<0.001
Cannabis Use Episodes Previous 6 Months	29 (±69)^b,c^	167 (±186)^a^	185 (±258)^a^	<0.001
Days since last cannabis use	26 (±43)	10 (±21)	51 (±153)	0.073
Nicotine use episodes previous 6 months		4 (±6)	1,512 (±2,278)	<0.001
Age of onset of nicotine use		16.58 (±2.24)	16.55 (±1.88)	>0.9
Years of nicotine use		3.17 (±2.60)	3.29 (±1.93)	0.7
Days since last nicotine use		253 (±538)	3 (±6)	<0.001
Number of cigarettes previous 6 months		3 (±3)	125 (±521)	0.3

Superscript letters denote significant group differences (*p* < 0.05). ^1^Mean (±SD); n (%). ^2^One-way ANOVA; Pearson’s Chi-squared test.

### 3.2 Hippocampal volume estimates

**Figure 1:**
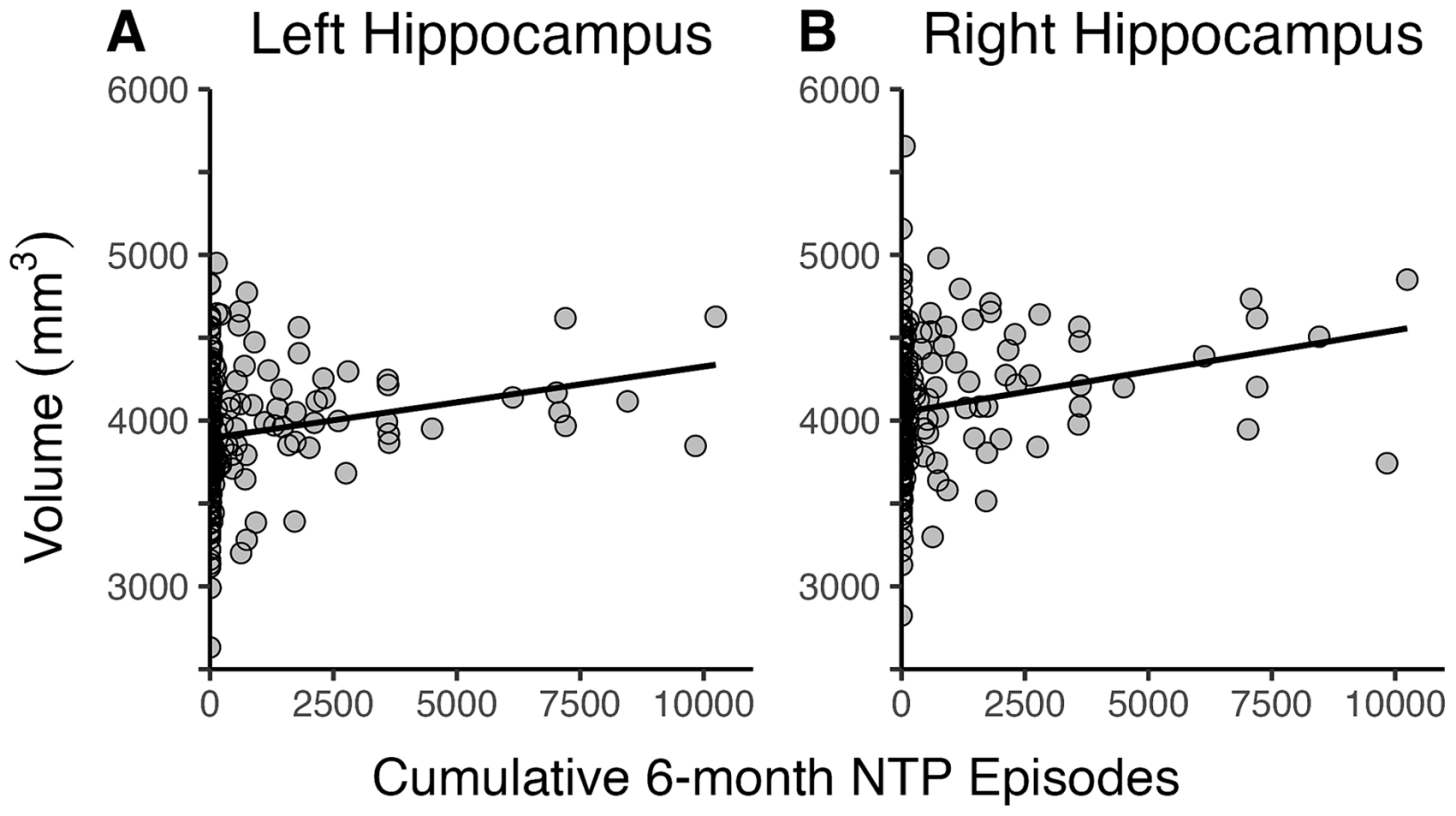
Scatterplots depicting relationship between cumulative 6-month NTP use and bilateral hippocampal volumes. Greater cumulative 6-month nicotine and tobacco product (NTP) use was associated with larger left **(A)** and right **(B)** hippocampal volumes.

Regression models examined the linear contributions of cumulative NTP and cannabis use episodes and their interaction on bilateral hippocampal volumes, controlling for age, sex, past 30-day alcohol use, and estimated brain volume. The overall models were significant for both left, *F*(7,213) = 27.5, *p* < 0.0001, *R*^2^ = 0.46, and right hippocampal volumes, *F*(7,213) = 24.4, *p* < 0.0001, *R*^2^ = 0.43. As seen in Figure 1, results indicated that greater NTP use episodes in the past 6 months predicted larger hippocampal brain volumes bilaterally (Left: *B* = 0.036, *t* = 2.4, *p* = 0.017; Right: *B* = 0.042, *t* = 2.6, *p* = 0.011), while cannabis use episodes had no significant impact (*p*’s > 0.2) nor was there an interaction between NTP and cannabis use (*p*’s > 0.1). Age, alcohol use, and sex were not significant covariates for either model (*p*’s > 0.2); however, estimated brain volume was a significant covariate for both (Left: *B* = 0.002, *t* = 9.8, *p* < 0.0001; Right: *B* = 0.002, *t* = 9.4, *p* < 0.0001). NTP age of initiation and recency of use were then explored as possible moderators within participants who reported lifetime NTP use. Neither significantly influenced the relationship between NTP use and hippocampal volumes (age of initiation: *p*’s > 0.08; recency: *p*’s > 0.3). Likewise, the possible contribution of combustible cigarette usage was explored in follow-up analyses; however, no significant association with hippocampal volume or NTP status by combustible product use interaction on volume was detected (*p*’s > 0.4).

Data from four participants was found to be highly influential for both the left and right hippocampi models with DFBETAS exceeding the threshold of 0.135. Models were rerun without these data points, and the results indicated that NTP use was still a significant predictor of bilateral hippocampal volumes (*p*’s < 0.05).

### 3.3 Relationship between hippocampal volume, NTP use, and memory performances

#### 3.3.1 NIH toolbox PSMT

**Figure 2:**
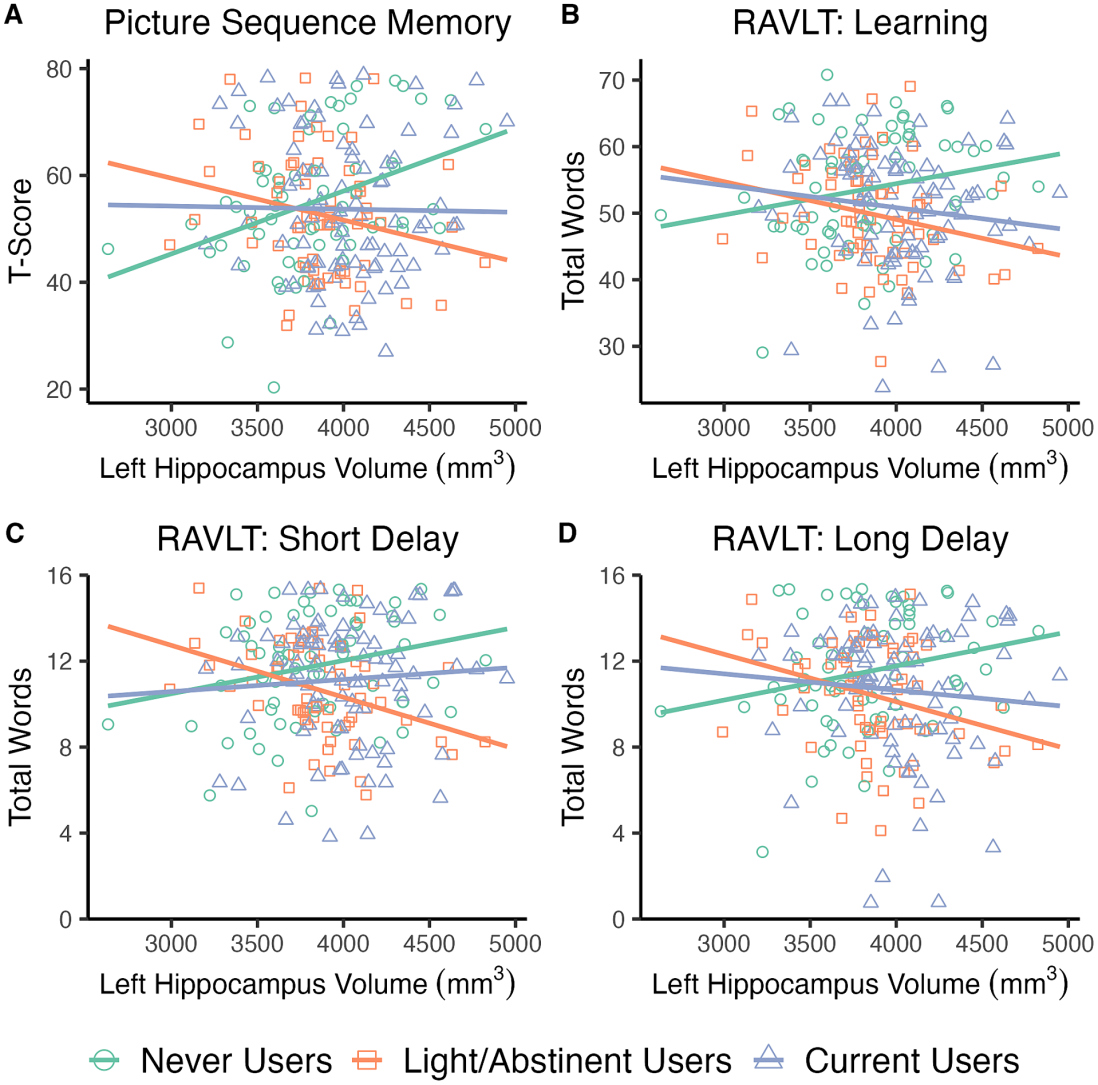
Scatterplots depicting relationship between nicotine group, hippocampal volume, and memory performance. Larger left hippocampal volumes predicted better performance for the Never Users on Picture Sequence Memory Test **(A)**, Rey Auditory Verbal Learning Test (RAVLT) total words learned across all 5 trials **(B)**, RAVLT short delay recall **(C)**, and RAVLT long delay recall **(D)**. In contrast, larger left hippocampal volumes were associated with worse performance for the Light/Abstinent Users across all measures. Larger left hippocampal volumes similarly predicted worse performance for Current Users for the Picture Sequence Memory Test, RAVLT total words, and a trend for long delay. Current Users did not differ from Never Users on short delay recall.

Individual linear regression models assessed the relationship between left and right hippocampus volumes and NTP group status on PSMT performance, controlling for crystalized composite score, past 6-month cumulative cannabis use, and past 30-day cumulative alcohol use. Significant Hippocampus Volume × NTP group interactions were found for both the Left, *F*(2,208) = 5.3, *p* = 0.006, and Right, *F*(2,208) = 3.2, *p* = 0.044, hippocampi (see Figure 2A; right not shown). Follow-up analyses indicated larger hippocampal volumes were positively associated with PSMT specifically for the NU group (Left: *B* = 0.012, *t* = 2.9, *p* = 0.004; Right: *B* = 0.009, *t* = 2.3, *p* = 0.023) while significant negative relationships were observed for LU (Left: *B* = −0.019, *t* = −3.2, *p* = 0.002; Right: *B* = −0.014, *t* = −2.3, *p* = 0.020) and CU groups, though the right was only at trend level (Left: *B* = −0.012, *t* = −2.1, *p* = 0.035; Right: *B* = −0.010, *t* = −2.0, *p* = 0.051). LU and CU groups did not differ from one another (*p* > 0.2). Crystalized composite scores were a significant covariate (Left: *B* = 0.233, *t* = 2.6, *p* = 0.009; Right: *B* = 0.240, *t* = 2.7, *p* = 0.008), but neither alcohol nor cannabis use were significant (*p*’s > 0.3).

#### 3.3.2 RAVLT learning, short delay, and long delay

##### 3.3.2.1 Verbal learning

Models indicated a significant Left Hippocampus Volume x NTP group interaction, *F*(2,205) = 3.2, *p* = 0.043, while a Right × NTP group interaction was not significant (*p* > 0.3). Follow-up analyses revealed that larger left hippocampal volume was a significant predictor of greater verbal learning (*B* = 0.006, *t* = 2.2, *p* = 0.030) for NU (Figure 2B). In contrast, larger left hippocampal volumes were associated with worse verbal learning for LU (*B* = −0.008, *t* = −2.2, *p* = 0.031) and CU (*B* = −0.007, *t* = −2.1, *p* = 0.036). No differences were detected between LU and CU (*p* > 0.8). This was after accounting for crystalized composite score (*B* = 0.411, *t* = 17.7, *p* < 0.001), biological sex (female as reference group: *B* = −2.4, *t* = −2.0, *p* = 0.046), and 6-month cannabis use episodes (*B* = −0.010, *t* = −3.5, *p* < 0.001). Age and alcohol use were not significant contributors (*p*’s > 0.09).

##### 3.3.2.2 Short delay recall

Hippocampal volume differentially contributed to verbal recall after a short delay depending on NTP group as indicated by a significant Left Hippocampus x NTP group interaction, *F*(2,205) = 4.4, *p* = 0.013. The Right Hippocampus × NTP group interaction was not significant (*p* > 0.4). For the NU group, larger left hippocampal volumes predicted greater verbal recall (*B* = 0.001, *t* = 1.9, *p* = 0.056; Figure 2C), while the larger left hippocampal volume was associated with worse performance for the LU group (*B* = −0.003, *t* = −2.9, *p* = 0.004). For the CU group, a significant difference was detected relative to the LU group (*B* = 0.001, *t* = 2.1, *p* = 0.036), while no differences were observed compared to the NU group (*p* > 0.3). Crystalized composite (*B* = 0.088, *t* = 3.2, *p* = 0.002) and cannabis use (*B* = −0.002, *t* = −2.8, *p* = 0.005) were significant covariates, while age, sex, and alcohol use were not (*p*’s > 0.07).

##### 3.3.2.3 Long delay recall

Finally, a similar significant Left Hippocampus × NTP group interaction was found for the number of words recalled after a long delay, *F*(2,205) = 3.2, *p* = 0.044, while no interaction was detected for the Right (*p* > 0.1). Follow-up analyses indicated the NU group again exhibited a positive relationship between hippocampal volume and the words recalled (*B* = 0.002, *t* = 2.0, *p* = 0.052; Figure 2D). In contrast, larger hippocampal volumes negatively predicted performance for LU (*B* = −0.003, *t* = −2.4, *p* = 0.018) with a similar trend for CU (*B* = −0.002, *t* = −1.8, *p* = 0.073). No differences were observed between CU and LU (*p* > 0.4). Crystalized composite (*B* = 0.101, *t* = 3.3, *p* = 0.001) and cannabis use (*B* = −0.003, *t* = −2.9, *p* = 0.004) were significant covariates, while age, sex, and alcohol use were not (*p*’s > 0.1).

## 4 Discussion

In the present study, we examined associations between cumulative 6-month NTP use and bilateral hippocampal volume in the context of cumulative 6-month cannabis use in a sample of late adolescents/young adults. The results indicated that greater NTP use predicted *larger* bilateral hippocampal volumes, while co-use of cannabis had no significant impact. We then examined whether NTP use at the group level modulated the relationship between hippocampal volumes and measures of learning and memory. We found differential associations between brain and cognitive performances such that larger hippocampal volumes, particularly left, were associated with better learning and memory for individuals who had never used NTPs, while larger volumes for adolescents/young adults who had used NTPs were linked with relatively lower memory scores.

Contrary to our hypothesis, cumulative NTP use was associated with *larger* hippocampal volumes. Previous studies examining structural differences in combustible tobacco-using adolescents and young adults have found *smaller* volumes (Harper et al., 2023) or no differences (Filbey et al., 2015) compared to non-using controls. Notably, however, these studies were with participants that were generally older and in their early- to mid-20s compared to the present study where the average age was ~19. Not only could these studies represent a different point in neurodevelopment (Spear, 2013), being older provides the opportunity for more years of nicotine use that could then be associated with greater cumulative effects (Leslie, 2020). Finally, use of traditional combustible cigarettes may have the potential for greater toxic exposure and addiction severity (Rubinstein et al., 2018; Marques et al., 2021; Lin et al., 2022; Wade et al., 2022). While use of combustible cigarettes did not moderate the relationship between NTP use and hippocampal volumes in the present study, the vast majority of our 16-22-year-old participants were e-cigarette users as compared to previous studies in which the samples were primary combustible users. Overall, differences in sample characteristics between the present study and those that have previously examined hippocampal volumes could provide insight into more subtle effects of NTP use, e-cigarette use in particular, during late adolescent/young adult neurodevelopment.

Nicotine exerts its effects on the brain by binding to nAChRs, which are abundant in the hippocampus (Zeid et al., 2018). Nicotinic activation of nAChRs can affect neuroimmune function by modifying microglia activity (Mahajan et al., 2021). Microglia are essential for synaptic pruning and cortical refinement during neurodevelopment (Paolicelli et al., 2011). In this context, the increased hippocampal volumes observed in the present study may represent nicotine-induced alterations in developmental trajectories, although prospective studies are needed to establish the directionality of effects. The impact on microglia may also contribute to nicotine’s neurotoxic effects on neuronal structure and integrity (Mahajan et al., 2021). In animal models of adolescent nicotine exposure, nicotine was associated with an increase in biomarkers indicative of decreased cell numbers but also with markers of increased cell size within the hippocampus such that gross anatomical weight of the region remained the same (Trauth et al., 2000; Abreu-Villaça et al., 2003; Oliveira-da-Silva et al., 2009). Notably, these changes occurred not only at blood plasma nicotine levels like could be found in current smokers but even at levels similar to brief intermittent exposure, highlighting that the adolescent brain is uniquely sensitive to nicotine.

In the present study, larger hippocampal volumes, particularly left, predicted worse verbal learning and memory performances for adolescents/young adults who had used NTPs (i.e., both Light/Abstinent Users and Current Users), while larger volumes were associated with better performance for the Never User group. Verbal learning and memory has largely been demonstrated to be preferentially subserved by the left hippocampus (Frisk and Milner, 1990; Lee et al., 2002; Pryor and Veselis, 2006; Sweatt, 2010), while more spatially-oriented memory with the right (Burgess et al., 2002). In this context, left hippocampal volumes being associated with better performance on the RAVLT for NTP Never Users is consistent with the broader literature. Moreover, a similar pattern was observed for bilateral hippocampal volumes predicting better NIH PSMT performance for Never Users with it additionally tapping into spatially memory. Importantly, the general pattern of relationships between hippocampal volumes and memory performances suggests that the morphological changes within the hippocampus associated with even light nicotine use may confer a functional disadvantage. NTP use during adolescence/young adulthood has consistently been linked to impairments on hippocampal-related cognitive processes (Jacobsen et al., 2005, 2007a,b; Colby et al., 2010; Filbey et al., 2015; Treur et al., 2015; Wade et al., 2021; Dai et al., 2022) as well as alterations in hippocampal activation (Jacobsen et al., 2007a,b; Rubinstein et al., 2011; Chen et al., 2018). As noted, nicotine has neurotoxic effects on neuronal structure and integrity within the hippocampus (Trauth et al., 2000; Abreu-Villaça et al., 2003; Oliveira-da-Silva et al., 2009). Animal studies have further demonstrated that adolescent nicotine exposure impacts neuritic projections and glial densities within the hippocampus (Abreu-Villaça et al., 2003; Oliveira-da-Silva et al., 2009), which could impact neuronal connections within the hippocampus and with other regions leading to functional detriment. Indeed, several recent human DTI studies have found nicotine use during adolescence/young adulthood to be associated with alterations in white matter morphometry (Courtney et al., 2022; Wallace et al., 2024). Notably, one such finding was within the fornix, a major output tract of the hippocampus, which could contribute to overall neural inefficiency during memory and learning. Overall, nicotine use during adolescence/young adulthood appears to have a negative relationship with hippocampal morphology with potential implications for downstream behavior.

Nicotine is commonly co-used with cannabis (Knapp et al., 2019; Moustafa et al., 2022), and we sought to examine the impact of nicotine use in this context. However, we found no relationship between cannabis use and hippocampal volumes nor was there an interaction between nicotine and cannabis use. This is contrary to expectation as meta-analyses suggest that regular cannabis use may be associated with reductions in hippocampal volume (Lorenzetti et al., 2019). However, this could represent opposing effects within individuals who moderately use both nicotine and cannabis (Courtney et al., 2022). That is, nicotine could be associated with relative increases in hippocampal volume while cannabis could be associated with decreases. As the majority of the present sample used both cannabis and nicotine at least minimally, these countering effects could obscure any potential interactions, particularly at lower levels of use. Consistent with previous studies (Scott et al., 2018; Jacobus et al., 2019), we did find cannabis use to be associated with poorer performance on measures of verbal learning and memory.

The results and conclusions of this study must be considered within its limitations. The study was cross-sectional in design, which limits the ability to make causal interpretations related to nicotine use. Differences in hippocampal brain volumes could have existed prior to nicotine initiation. Though beyond the scope of this study, there is growing evidence that multigenerational substance use may contribute to brain development and influence baseline morphometry (Cservenka, 2016; Henderson et al., 2018; Gonçalves et al., 2024). Therefore, large, longitudinal studies such as the Adolescent Brain Cognitive Development (ABCD) Study (Volkow et al., 2018) that examine and follow adolescents prior to and after initiation of nicotine use will be essential for determining relationships between nicotine and brain health as well as the contributions of transgenerational substance use to developmental trajectories. Additionally, while alcohol was controlled for in this study and was not a significant covariate, alcohol could still have an impact on brain development, including the hippocampus (Jacobus and Tapert, 2013; Squeglia et al., 2014; Tapert and Eberson-Shumate, 2022). Large studies such as ABCD will be well-powered to assess the impact of polysubstance use (e.g., co-use of alcohol with NTPs and cannabis) on brain morphometry. We also failed to find any relationship between hippocampal volumes and cannabis use, contrary to the extant literature (Lorenzetti et al., 2019), although a large percentage of our sample report use of both cannabis and nicotine. Thus, our findings may not align with existing studies that focus on individuals who engage in single substance cannabis use only.

In sum, the present study examined changes in hippocampal morphometry and function associated with NTP use in a sample of adolescent/young adults. The results indicate that greater nicotine use predicted increased bilateral hippocampal volume which could represent alterations in neurodevelopmental trajectories. Importantly, larger volumes in adolescents/young adults who had ever used NTPs were associated with worse performance on cognitive processes dependent on hippocampal integrity. While these findings were examined in the context of cannabis co-use, no interaction between NTP and cannabis nor an effect of cannabis alone was detected, though this could suggest opposing effects of the two substances. Greater understanding of the impact of nicotine use on brain health during this vulnerable developmental period are necessary for guiding public health policy.

## Data Availability

The raw data supporting the conclusions of this article will be made available by the authors, without undue reservation.
